# Tau in Atypical Parkinsonisms: A Meta‐Analysis of in Vivo PET Imaging Findings

**DOI:** 10.1002/mdc3.13885

**Published:** 2023-09-29

**Authors:** Anastassia M. Mena, Robert Chen, Ariel Graff‐Guerrero, Sarah L. Martin, Carme Uribe, Antonio P. Strafella

**Affiliations:** ^1^ Brain Health Imaging Centre, Campbell Family Mental Health Research Institute, Centre for Addiction and Mental Health University of Toronto Toronto Ontario Canada; ^2^ Institute of Medical Science University of Toronto Toronto Ontario Canada; ^3^ Division of Brain, Imaging and Behaviour–Systems Neuroscience, Krembil Brain Institute, UHN University of Toronto Toronto Ontario Canada; ^4^ Edmond J. Safra Parkinson Disease Program & Morton and Gloria Shulman Movement Disorder Unit, Neurology Division, Department of Medicine, Toronto Western Hospital, UHN University of Toronto Toronto Ontario Canada

**Keywords:** parkinsonism, PSP, CBD, MSA, Parkinson's disease, neuroimaging, PET, SPECT

## Abstract

**Background:**

Progressive supranuclear palsy (PSP) and corticobasal degeneration (CBD) are atypical parkinsonisms (APs) that are classified as tauopathies. Patients with these APs may present with similar early clinical manifestations to Parkinson's disease (PD), but they prove unresponsive to anti‐parkinsonian medications.

**Objective:**

The main objective of this meta‐analysis was to compare first‐ and second‐generation tau PET tracer efficacy in patients with the APs to identify potential diagnostic biomarkers.

**Methods:**

PubMed and Web of Science were searched between January 1, 1999 and December 31, 2022. We included case–control studies that were published in English and report tau PET tracer binding as mean ± SD in at least one region of interest (ROI). Differences in tau PET binding values were meta‐analyzed using random‐effects meta‐analytic models and subgroup analyses based on ROIs in the statistical programming language R (version 4.2.1).

**Results:**

Overall, 29 studies with 665 patients were included in the final review. [^18^F]PI‐2620 outperformed first‐generation tracers when comparing PSP‐HC (*g* = −1.68, 95% CI: −2.05 to −1.30) and CBD‐HC (*g* = −1.37, 95% CI: −2.25 to −0.49). When comparing PSP‐PD, the first‐generation tracer, [^18^F]AV‐1451, presented with higher binding to PSP patients (*g* = −0.80, 95% CI: −1.24 to −0.35).

**Conclusions:**

Our results demonstrate the efficacy of [^18^F]PI‐2620 PET in imaging AP‐tau. These findings contribute towards identifying a diagnostic imaging biomarker for patients with APs. The main limitation of this study was the heterogeneity of the results. Future studies should conduct AP‐PD comparisons with second‐generation tracers to confirm the preliminary results found here.

Atypical parkinsonisms (APs) are a group of rare neurodegenerative disorders that present with heterogeneous symptomology and neuropathology. Since many of these disorders present with similar symptomology early on in disease process, one of the innovative ways that researchers and clinicians distinguish APs is through imaging the pathological protein deposits present within various brain regions. For Parkinson's disease (PD), the neuropathology is predominantly characterized by deposition of the alpha‐synuclein protein.^1^, ^2^ An example of an AP alpha‐synucleinopathy is multiple system atrophy (MSA). Another group of AP proteinopathies are the tauopathies, which include progressive supranuclear palsy (PSP) and corticobasal degeneration (CBD). Although these APs are categorized according to the pathological protein that predominates, it is important to note that we sometimes see pathological tau in both PD and MSA patients albeit to a lesser degree than alpha‐synuclein, thus introducing another concept of mixed pathologies.^3^, ^4^

Under normal physiological conditions, tau functions as a microtubule‐associated protein (MAP) that promotes microtubule assembly and aids in microtubule stability, especially in the neurons of the central nervous system (CNS).^5^, ^6^ Under pathological conditions, the tau protein becomes hyperphosphorylated and forms insoluble tau aggregates called neurofibrillary tangles (NFTs) that aggregate in a variety of different brain regions. Previous literature posits that the deposition of these tau NFTs is closely linked to cognitive decline in Alzheimer's disease (AD) and other tauopathies.^7^ For this reason, quantifying tau is an important step in biomarker development progress, particularly for PSP and CBD where there are few other early indicators for their diagnosis.

One avenue of biomarker development has been the use of molecular imaging, particularly positron emission tomography (PET) imaging. PET imaging is a useful tool for the localization of tau because of the development of tau PET radiotracers that bind to the microtubule binding domain of tau.^8^, ^9^ The inception of tau PET imaging began with the development of the [^18^F]AV‐1451 tracer. This tracer was recently approved by the United States Food and Drug Administration for effective use with imaging AD‐tau.^10^ Following the development of this tracer, there were a variety of other first‐generation tracers developed including [^18^F]THK‐5351 and [^11^C]PBB3. As summarized in our previously published systematic review,^11^ these first‐generation tracers demonstrate limited specificity and capacity to localize PSP‐ and CBD‐tau. For this reason, recent work has focused on the performance of the second‐generation tau PET tracers. These include [^18^F]PI‐2620, [^18^F]PM‐PBB3, and [^18^F]MK‐6240. Although the literature points towards the improved clinical utility of the second‐generation tracers, there has yet to be a quantitative analysis of the performance of first‐ versus second‐generation tracers.

The present paper aims to meta‐analyze tau PET tracer binding values in PSP, CBD, and PD patients using pooled PET imaging studies’ data. We aimed to determine (1) which tracers preferentially bind to pathological patients over healthy controls (HC), (2) which tracers can distinguish parkinsonisms, (3) which regions of interest (ROIs) demonstrate the highest tracer binding, and (4) if there are any confounding variables that influence tracer binding.

## Methods

The methodology for this study was carried out in accordance with the Preferred Reporting Items for Systematic Reviews and Meta‐Analysis (PRISMA) guidelines.^12^

### Search Strategy

PubMed and Web of Science were searched between January 1, 1999 and December 31, 2022 for publications studying tau PET tracers in vivo in patients with APs and PD. Additionally, the references of the resulting articles and review papers were manually searched for missing studies.

### Study Selection

**Figure 1:**
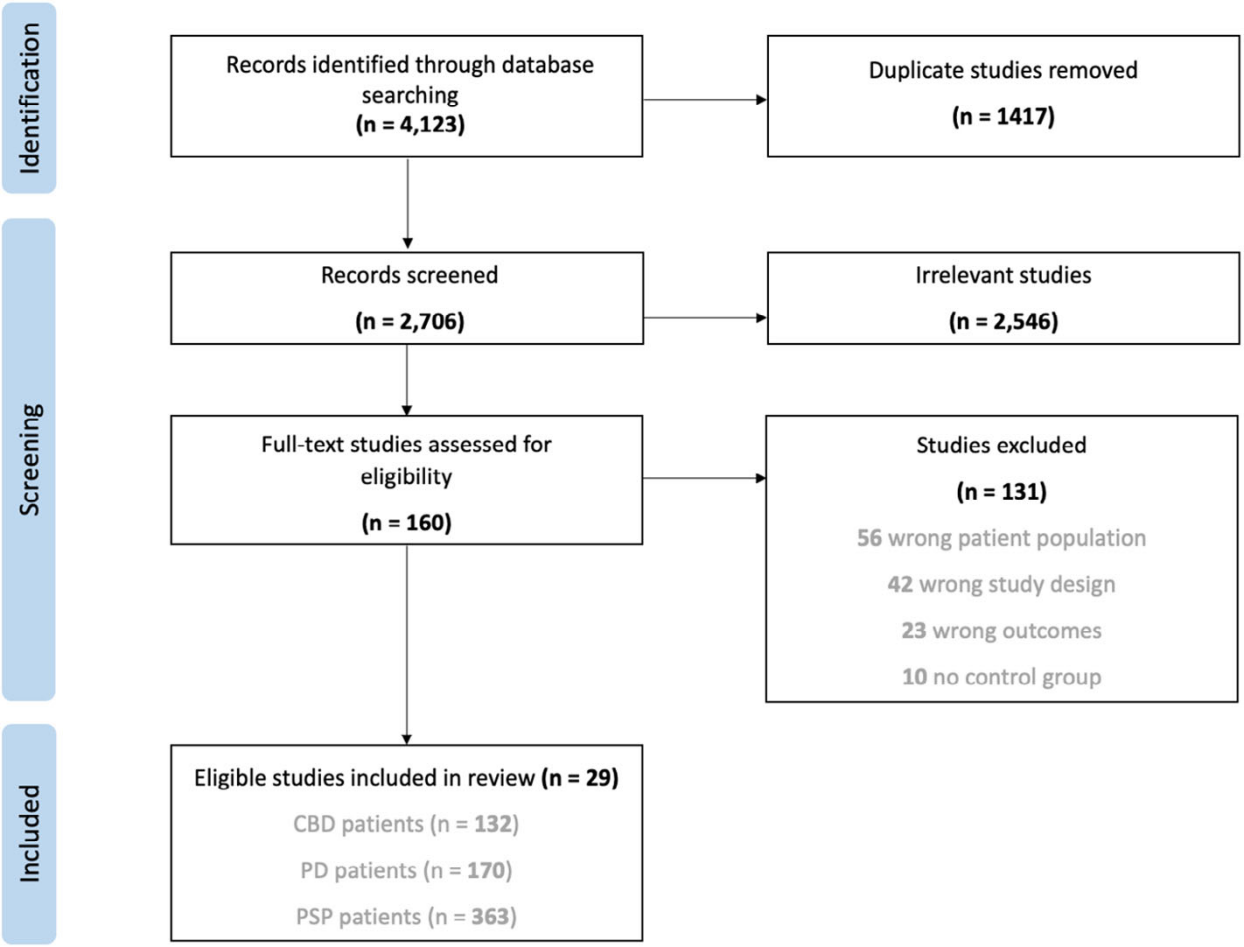
Preferred reporting items for systematic reviews and meta‐analysis (PRISMA) flow diagram.

Using an internet‐based software called Covidence (www.covidence.org), we conducted study screening and data extraction (Fig. 1). Initial screening included abstract and title reviews. Following this, eligible studies underwent full‐text reviews against our inclusion and exclusion criteria. Finally, unanimously approved studies were moved into data extraction.

The included studies meet the following criteria: (1) published in English; (2) involve human tau PET imaging; (3) assess tau PET tracer binding; (4) compare PD, PSP, CBD, or MSA patients to HC or APs to PD; and (5) report tau PET tracer binding as mean ± SD in at least one ROI.

### Study Quality Assessments

All studies were assessed for publication bias using funnel plots and trim‐and‐fill analyses with imputed data points in addition to Egger's test for asymmetry. Study quality was evaluated using a modified Newcastle‐Ottawa scale (NOS) (Table S1). Each study was assessed against the criteria and received a score of 0–6 stars.

### Data Extraction

The variables extracted were first author's surname, publication year, binding uptake calculation method, number of subjects in each participant group, mean ± SD age (years), sex of participants, mean ± SD disease duration (years), mean ± SD Unified Parkinson's Disease Rating Scale (UPDRS) motor score, mean ± SD Hoehn and Yahr (HY) stage score, mean ± SD Progressive Supranuclear Palsy Rating Scale (PSPRS) score, mean ± SD Cortical Basal ganglia Functional Scale (CBFS) scores, scanner type, mean injected tracer dose (MBq), scan duration (minutes), and mean ± SD uptake data for each participant group and brain region.

### Data Analysis

All statistical analyses were carried out using the “meta” (version 6.0.0) and “metafor” (version 3.8.1) packages in the statistical programming language R (version 4.2.1). Meta‐analyses were conducted separately for all group comparisons (ie, PSP‐PD, PSP‐HC, CBD‐HC, PD‐HC) and only performed if at least three eligible studies were available. The meta‐analysis proper was conducted using a random‐effects model. These models meta‐analyze study effect sizes using restricted maximum likelihood estimation that reports study effect sizes as Hedge's g values representing the standardized mean difference (SMD) in tracer binding between participant groups. Heterogeneity of the effect sizes was assessed using *I* ^2^ statistics, where values below 30% represent low heterogeneity, between 40% and 60% represents moderate heterogeneity, and greater than 60% represents high heterogeneity. If substantial heterogeneity with *I* ^2^ > 50% was present in the results, we assessed the influence of potential clinical and demographic effect moderators on tracer uptake. These moderators include publication year, percentage of female participants, age, disease duration, UPDRS motor score, HY stage score, PSPRS score, and CBFS score. The effects of these variables were assessed using meta‐regression analyses.

In order to assess binding in the various ROIs set out for each tracer, we performed subgroup analyses between ROIs for each tau PET tracer using random effects models for between‐subgroup weighting. Comparison of the combined subgroup effect sizes was conducted using an analysis of variance based on sum of squares. A statistical significance level of *P* < 0.05 was used for all analyses.

The group comparison of PSP to PD only had sufficient data from [^18^F]AV‐1451 studies. Data were sufficient for comparison of PSP patients to HC with [^18^F]AV‐1451, [^18^F]THK‐5351, and [^18^F]PI‐2620. For the comparison of CBD patients to HC, there was sufficient data for [^18^F]AV‐1451 and [^18^F]PI‐2620. The first‐generation tracer [^18^F]AV‐1451 and second‐generation tracer [^18^F]PM‐PBB3 were analyzed for comparison of PD to HC. In this analysis, sufficient data is defined as having three or more studies assessing identical comparisons in terms of participant groups, tau PET tracers, and at least one ROI. Since the studies that included MSA did not have similar ROIs or consistent comparisons to any other groups assessed, they were excluded from the analysis.

### Data Quality

All studies scored ≥4 stars on the modified NOS (Table S1). Funnel plots with imputed datapoints for the PSP‐PD analysis revealed 0 missing studies ([^18^F]AV1451: *P* = 0.709) with trim‐and‐fill–adjusted effect sizes remaining the same, suggesting minimal impact of publication bias. Funnel plots for the PSP‐HC comparisons suggested some publication bias for studies with all three tracers assessed ([^18^F]AV‐1451: *P* = 0.01, [^18^F]THK‐5351: *P* = 0.006, [^18^F]PI‐2620: *P* = 0.038). For the [^18^F]AV‐1451 PSP‐HC analysis, trim‐and‐fill analysis revealed 1 positive missing study but the adjusted effect size remained essentially the same, suggesting limited publication bias. There were four positive studies missing for the [^18^F]THK‐5351 PSP‐HC analysis and the trim‐and‐fill adjusted effect size switched from negative to positive, indicating an influence of publication bias on the combined effect for studies with this tracer. Similarly, there were two positive studies missing for the [^18^F]PI‐2620 PSP‐HC analysis but in this instance the trim‐and‐fill adjusted effect size remained similar, indicating limited publication bias. The funnel plot for the [^18^F]AV‐1451 CBD‐HC comparisons suggests publication bias ([^18^F]AV‐1451: *P* < 0.001) but there is only one positive missing study and trim‐and‐fill adjusted effect sizes remain essentially the same, indicating minimal publication bias. Conversely, the funnel plot for the [^18^F]PI‐2620 CBD‐HC comparison suggests no significant publication bias with 0 studies missing and trim‐and‐fill adjusted effect sizes remaining the same ([^18^F]PI‐2620: *P* = 0.509). Funnel plots with imputed data points for the PD‐HC group comparisons suggested no significant publication bias with 0 studies missing and trim‐and‐fill adjusted effect sizes remaining the same ([^18^F]AV‐1451: *P* = 0.427, [^18^F]PM‐PBB3: *P* = 0.444).

## Results

### Study Characteristics

A summary of the included studies can be found in Table S2. The demographic and clinical characteristics of the participants of each study included can be found in Tables 1 and 2, respectively.

**TABLE 1:** Demographic characteristics of participants in included studies

Study	PD (n)	HC (n)	PSP (n)	CBD (n)	PD age (yrs)	PD sex (M/F)	HC age (yrs)	HC sex (M/F)	PSP age (yrs)	PSP sex (M/F)	CBD age (yrs)	CBD sex (M/F)
Hansen^13^	‐	7	7	‐	‐	‐	67	4/3	66	4/3	‐	‐
Cho^14^	15	15	14	‐	67.9 (5.4)	8/7	68.7 (5.3)	8/7	70.8 (6.2)	13/1	‐	‐
^15^	9	29	‐	‐	67 (3)	8/1	69 (2)	16/13	‐	‐	‐	‐
Holland^16^	‐	19	23	12	‐	‐	68.9 (7.1)	11/8	71.3 (8.6)	10/13	70.9 (7.9)	7/5
Whitwell^17^	‐	39	16	‐	‐	‐	‐	‐	68 (6) [59–83]	10/6	‐	‐
Smith^18^	‐	11	11	‐	‐	‐	70.9 (1.9)	‐	70.7 (2.2)	‐	‐	‐
Ossenkoppele^19^	23	160	40	23	67.3 (5.8) [56–76]	65.2% male	69.1 (0.5) [41–90]	40.6% male	69.9 (6.6) [57–85]	67.5% male	69.1 (6.3) [59–80]	52% male
Coakeley^20^	6	10	6	‐	63.67 (9.61)	3/3	65.9 (9.93)	2/8	72.2 (6.77)	2/4	‐	‐
Schonhaut^21^	26	46	33	‐	67.1 (5.4)	14/12	69.6 (5.4)	25/21	69.6 (5.7)	23/10	‐	‐
Li^22^	8	10	7	9	66.4 (5.7)	2/6	60.1 (10.4)	2/8	70.0 (6.5)	3/4	63.3 (13.2)	2/7
Niccolini^23^	‐	20	‐	11	‐	‐	72.4 (4.8)	10/10	‐	‐	69.2 (6.8)	5/6
Smith^54^	‐	17	11	‐	‐	‐	73 (6)	‐	71 (7)	‐	‐	‐
Tsai^24^	‐	53	‐	10	‐	‐	76 [20–93]	30/23	‐	‐	68 [54–77]	5/5
Winer^25^	15	25	‐	‐	66.6 (6.6)	7/8	71.7 (6.7)	11/14	‐	‐	‐	‐
Hansen^26^	17	23	‐	‐	67.6 (6.3)	13/4	68.6 (7.2)	15/8	‐	‐	‐	‐
Brendel^27^	‐	9	11	‐	‐	‐	71.6 (6.6)	5/4	68.4 (7.4)	5/6	‐	‐
Ezura^28^	‐	9	9	7	‐	‐	71.0 (6.44) [61–81]	5/4	74.1 (5.28) [66–81]	9/0	69.1 (4.85) [64–78]	2/5
Hsu^29^	‐	28	17	‐	‐	‐	66.2 (4.5)	10/18	68.8 (6.5)	9/8	‐	‐
Ishiki^30^	‐	9	3	‐	‐	‐	71.6 (6.6)	5/4	78.3 (3.7)	3/0	‐	‐
Ng^31^	‐	2	4	‐	‐	‐	64.5	1/1	78.5	3/1	‐	‐
Song^32^	‐	11	15	14	‐	‐	67.5 (6.7)	73% female	71.0 (7.2)	38% female	71.0 (7.2)	38% female
Messerschmidt^33^	‐	10	36	‐	‐	‐	67 (7)	2/8	71 (8)	20/16	‐	‐
Oh^34^	2	3	3	3	75.5	2/0	63.7	2/1	64.3	2/1	65.3	3/0
Palleis^35^	‐	14	‐	34	‐	‐	67.4 (9.5)	5/9	‐	‐	69.9 (7.7)	15/19
Song^36^	‐	10	37	‐	‐	‐	67.0 (7.4)	2/8	70.8 (6.3)	22/15	‐	‐
Brendel^37^	6	10	40	9	60 (10)	4/2	67 (7)	2/8	71 (6)	22/18	70 (11)	3/6
Li^38^	10	13	20	‐	59.4 (16.3)	6/4	60.6 (4.2)	8/5	63.0 (7.4)	13/7	‐	‐
Liu^55^	24	20	‐	‐	60.75 (14.14)	16/8	56.6 (7.11)	6/14	‐	‐	‐	‐
Tang^39^	9	9	‐	‐	69.2 (6.8)	6/3	63.8 (3.6)	4/5	‐	‐	‐	‐

Abbreviations: CBD, corticobasal degeneration; HC, healthy controls; PSP, progressive supranuclear palsy; PD, Parkinson's disease.

**TABLE 2:** Clinical characteristics of participants in included studies

Study	PD duration (yrs)	PD motor UPDRS	PD HY	PSP duration (yrs)	PSP CBFS	PSP PSPRS	PSP UPDRS	PSP HY	CBD duration (yrs)	CBD PSPRS	CBD CBFS	CBD UPDRS
Hansen^13^	5.2 [0.5–12.4]	23.9 (12.6)	2 [1–3]	‐	‐	‐			‐	‐	‐	
Cho^14^	5.5 (3.5)	26.9 (11.2)	‐	4.5 (3.1)	‐	32.6 (10.4)			‐	‐	‐	
Gomperts^15^	‐	23.3 (12.8)	‐	‐	‐	‐			‐	‐	‐	
Holland^16^	‐	‐	‐	3.9 (2.2)	32.7 (15.9)	32.7 (8.2)			3.9 (2.1)	28.9 (10.0)	26.2 (16.2)	
Whitwell^17^	‐	‐	‐	4.4 (1.7) [2.3, 8.9]	‐	36 (14) [9, 60]			‐	‐	‐	
Smith^18^	‐	‐	‐	5.27 (2.6)	‐	40 [24–64]		4 [2–5]	‐	‐	‐	
Ossenkoppele^19^	‐	‐	‐	‐	‐	‐	‐	‐	‐	‐	‐	‐
Coakeley^20^	5.50 (2.43)	26.3 (3.01)	‐	4.00 (1.41)	‐	45.0 (9.81)		‐	‐	‐	‐	
Schonhaut^21^	‐	26.1 (11.4)	‐	‐	‐	34.7 (11.6)	23.2 (10.1)	‐	‐	‐	‐	
Li^22^	8.1 (6.6)	18.1 (14.6)	‐	4.7 (3.0)	‐	‐	39.4 (18.3)		3.3 (1.0)	‐	70.0 (6.5)	30.1 (10.0)
Niccolini^23^	‐	‐	‐	‐	‐	‐	‐	‐	4.82 (2.2)	‐	‐	‐
Smith^54^	‐	‐	‐	‐	‐	‐	‐	‐	2.25	‐	‐	38.25
Tsai^24^	‐	‐	‐	‐	‐	‐	‐	‐	‐	‐	‐	‐
Winer^25^	‐	24.9 (12.7)	‐	‐	‐	‐	‐	‐	‐	‐	‐	‐
Hansen^26^	4.5 (3.2)	19.2 (8.4)	2.1 (0.6)	‐	‐	‐	‐	‐	‐	‐	‐	‐
Brendel^27^	‐	‐	‐	3 (1.58)	‐	30 [19–44]	‐	‐	‐	‐	‐	‐
Ezura^28^	‐	‐	‐	‐	‐	44.9 (19.4)	38.9 (14.6)		‐	34.0 (21.7)	‐	33.7 (18.3)
Hsu^29^	‐	‐	‐	6.6 (3.8)	‐	‐			‐	‐	‐	‐
Ishiki^30^	‐	‐	‐	‐	‐	‐	‐	‐	‐	‐	‐	‐
Ng^31^	‐	‐	‐	‐	‐	36.75	‐	‐	‐	‐	‐	‐
Song^32^	‐	‐	‐	‐	‐	‐	‐	‐	‐	‐	‐	‐
Messerschmidt^33^	‐	‐	‐	3.3 (2.6)	‐	31 (13)	‐	‐	‐	‐	‐	‐
Oh^34^	‐	‐	‐	‐	‐	‐	‐	‐	‐	‐	‐	‐
Palleis^35^	‐	‐	‐	‐	‐	‐	‐	‐	2.73 (1.61)	26.5 (13.3)	‐	‐
Song^36^	‐	‐	‐	‐	‐	‐	‐	‐	‐	‐	‐	‐
Brendel^37^	1.67 (1.42)	23.9 (6.2)	2.4 (0.8)	4.08 (3.17)	‐	37.2 (15.1)	‐	‐	3.5 (3.08)	26.2 (9.6)	‐	‐
Li^38^	2.49 (1.44)	38.9 (17.1)	4 [3–4]	3.76 (2.5)	‐	31.6 (16.6)	34.2 (15.1)	3 [3–3]	‐	‐	‐	‐
Liu^55^	1.75 [1.02–4.44]	43.13 (15.8)	3 [2–3]	‐	‐	‐	‐	‐	‐	‐	‐	‐
Tang^39^	2.02 (1.46)	40.8 (17.6)	2.4 (0.5)	‐	‐	‐	‐	‐	‐	‐	‐	‐

Abbreviations: CBD, corticobasal degeneration; CBFS, cortical basal ganglia functional scale; HC, healthy controls; HY, Hoehn and Yahr; PSP, progressive supranuclear palsy; PD, Parkinson's disease; PSPRS, progressive supranuclear palsy rating scale; UPDRS, Unified Parkinson's disease rating scale.

### Progressive Supranuclear Palsy

#### First‐Generation Tracers

**Figure 2:**
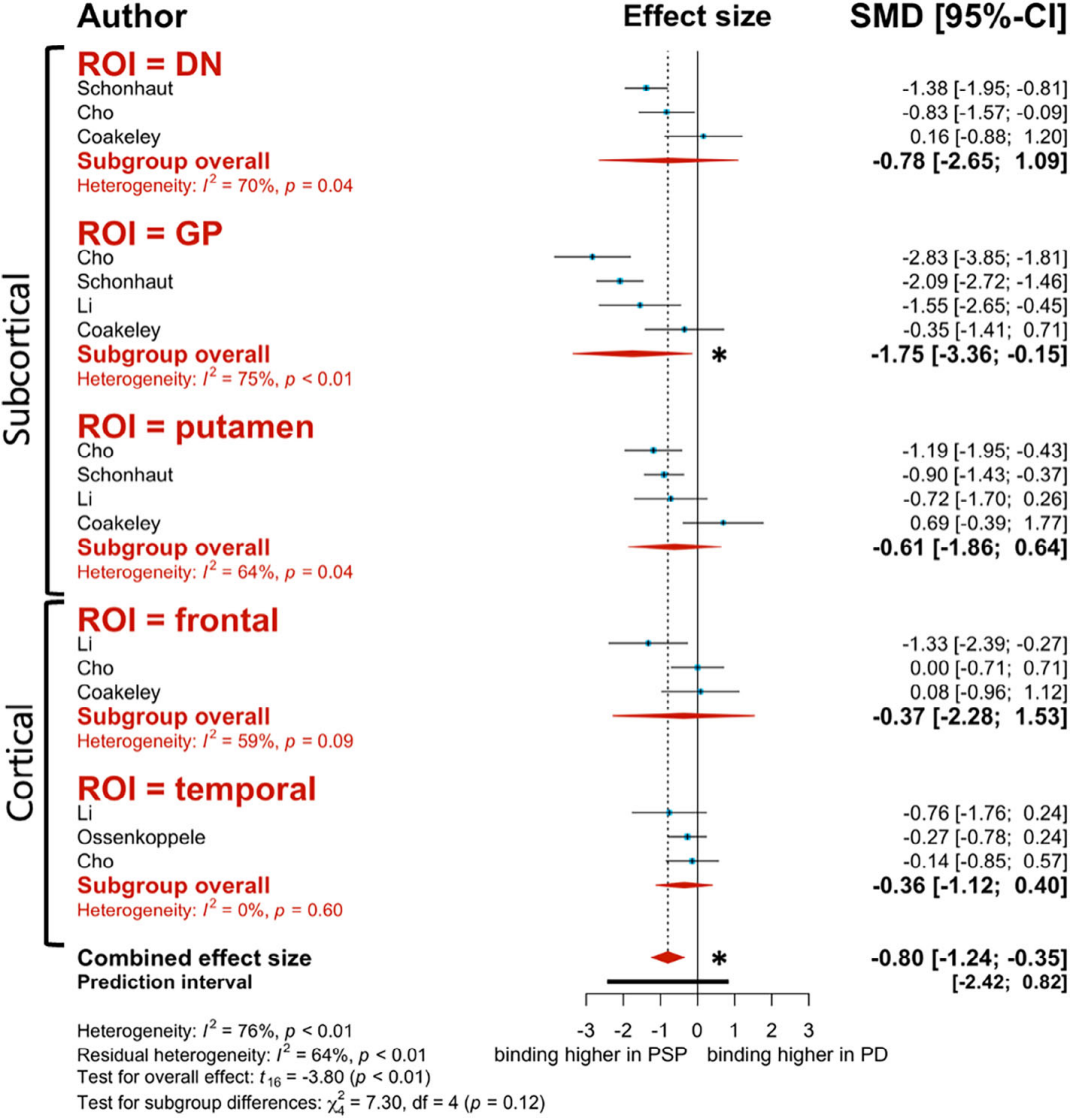
Forest plot of differences in first‐generation tracer [^18^F]AV‐1451 binding to progressive supranuclear palsy (PSP) versus Parkinson's disease (PD) patients. Red diamonds represent subgroup analysis based on region of interest (ROI). Blue data points represent individual study effect sizes. The * represents statistical significance (*P* < 0.05).

[^18^F]AV‐1451 tracer binding was overall significantly higher in *PSP patients compared to PD patients* (*P* = −0.80, 95% CI: −1.24 to −0.35, *P* = 0.002; Fig. 2). Although tracer binding in most ROIs trended towards higher binding in PSP patients, the only region that displayed statistical significance was the globus pallidus (GP) (*g* = −1.75, 95% CI: −3.36 to −0.15, *I* ^2^ = 75.2%; Fig. 2). Similarly, [^18^F]AV‐1451 displayed significantly higher binding to the *PSP participants over HC* (*g* = −1.01, 95% CI: −1.46 to −0.57, *P* < 0.0001). We observed this trend in tracer binding for all ROIs assessed, except the temporal lobe (*g* = 0.28, 95% CI: −1.35 to 1.91, *I* ^2^ = 79.8%). Notably, the ROIs that displayed significantly higher binding in PSP participants over HC were the dentate nucleus (DN) (*g* = −0.95, 95% CI: −1.89 to −0.01, *I* ^2^ = 68.2%), GP (*g* = −2.13, 95% CI: −3.22 to −1.03, *I* ^2^ = 81.3%), and putamen (*g* = −1.01 95% CI: −1.97 to −0.05, *I* ^2^ = 72.9%).

Meta‐regression analysis revealed that PSP duration was an effect moderator for the PSP‐PD analysis. Greater PSP duration was associated with greater binding to PSP participants over PD (estimate = −1.859, 95% CI: −3.13 to −0.586, *P* = 0.007). Interestingly, age was also a significant effect moderator. Surprisingly and contrary to what we expected, aging among study participants correlated with decreased binding in PSP relative to PD (estimate = 0.455, 95% CI: 0.0857 to 0.823, *P* = 0.018), suggesting a potential limiting/confounding factor related to this radiotracer.

Similar performance was observed for [^18^F]THK‐5351, where we found significantly higher binding in the *PSP patients compared to HC* overall (*g* = −1.05, 95% CI: −1.74 to −0.37, *P* = 0.006) and particularly in the GP (*g* = −1.55, 95% CI: −2.99 to −0.10, *I* ^2^ = 75.3%). Although the GP was the only ROI that demonstrated statistical significance, both the putamen (*g* = −0.42, 95% CI: −0.83 to −0.002, *I* ^2^ = 0.0%) and frontal lobe (*g* = −1.15, 95% CI: −3.59 to 1.29, *I* ^2^ = 87.5%) presented with a trend towards higher binding in PSP patients over HC. For the [^18^F]THK‐5351 meta‐regression analysis, publication year and PSP duration were significant effect moderators. Interestingly, more recent publications with this tracer presented with decreased binding in PSP over HC (estimate = 0.4299, 95% CI: 0.0077 to 0.852, *P* = 0.0464). Another surprising observation was that longer PSP durations were similarly associated with decreased binding to PSP (estimate = 0.684, 95% CI: 0.124 to 1.244, *P* = 0.0244), suggesting also in this case a potential limiting/confounding factor related to this radiotracer.

#### Second‐Generation Tracer

**Figure 3:**
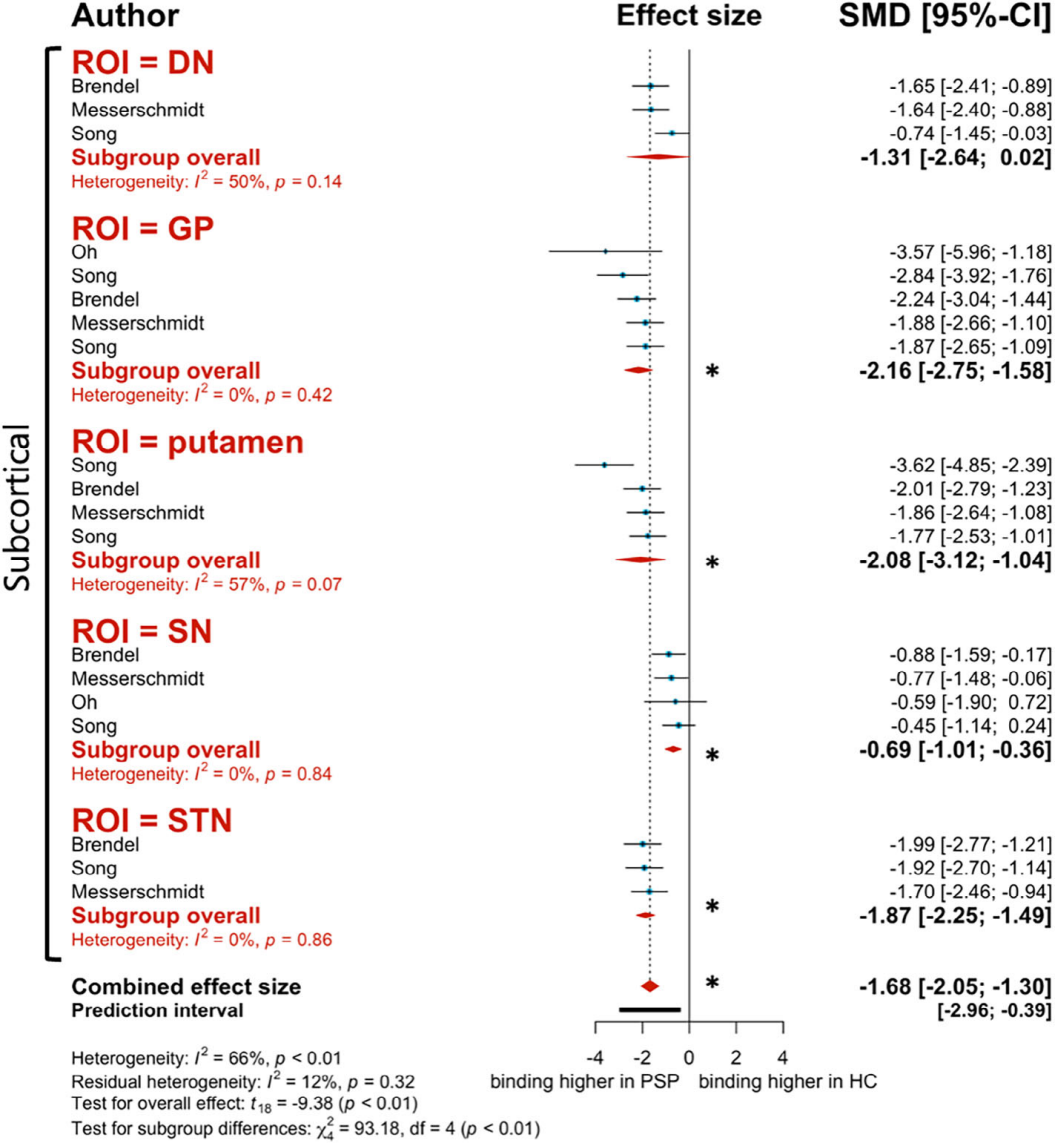
Forest plot of differences in second‐generation tracer [^18^F]PI‐2620 binding to progressive supranuclear palsy (PSP) patients versus healthy controls (HC). Red diamonds represent subgroup analysis based on region of interest (ROI). Blue data points represent individual study effect sizes. The * represents statistical significance (*P* < 0.05).

Like the first‐generation tracers described above, the second‐generation tracer [^18^F]PI‐2620 demonstrated significantly higher binding in *PSP patients when compared to HC* (*g* = −1.68, 95% CI: −2.05 to −1.30, *P* < 0.0001; Fig. 3). This significant trend is consistent throughout most ROIs assessed in this analysis, including the GP (*g* = −2.16, 95% CI: −2.75 to −1.58, *I* ^2^ = 0.0%), putamen (*g* = −2.08, 95% CI: −3.12 to −1.04, *I* ^2^ = 56.8%), substantia nigra (SN) (*g* = −0.69, 95% CI: −1.01 to −0.36, *I* ^2^ = 0.0%), and subthalamic nucleus (STN) (*g* = −1.87, 95% CI: −2.25 to −1.49, *I* ^2^ = 0.0%) (Fig. 3). Although not statistically significant, binding to the DN still presented with a tendency to be higher for PSP patients (*g* = −1.31, 95% CI: −2.64 to 0.02, *I* ^2^ = 49.8%; Fig. 3). Meta‐regression analysis revealed no significant effect moderators.

### Corticobasal Degeneration

#### First‐Generation Tracers

When *comparing CBD patients to HC*, there was an overall trend towards higher [^18^F]AV‐1451 binding in CBD patients (*g* = −1.00, 95% CI: −2.00 to 0.01, *P* = 0.05). Although none of the ROIs assessed displayed statistical significance, the combined effect size and all ROI subgroups present negative values, indicating a trend to higher binding in the CBD patients over HC. Meta‐regression analysis revealed publication year, disease duration, and age as effect moderators for the meta‐analysis with contrasting results. More recent publications were associated with higher binding in HC (estimate = 0.731, 95% CI: 0.227 to 1.235, *P* = 0.007); and studies with older CBD participants demonstrated higher binding to CBD patients over HC (estimate = −0.093, 95% CI: −0.183 to −0.003, *P* = 0.0434). However, contrary to what we expected, increased CBD duration was associated with decreased binding in CBD participants (estimate = 1.299, 95% CI: 0.401 to 2.197, *P* = 0.0078).

#### Second‐Generation Tracer

**Figure 4:**
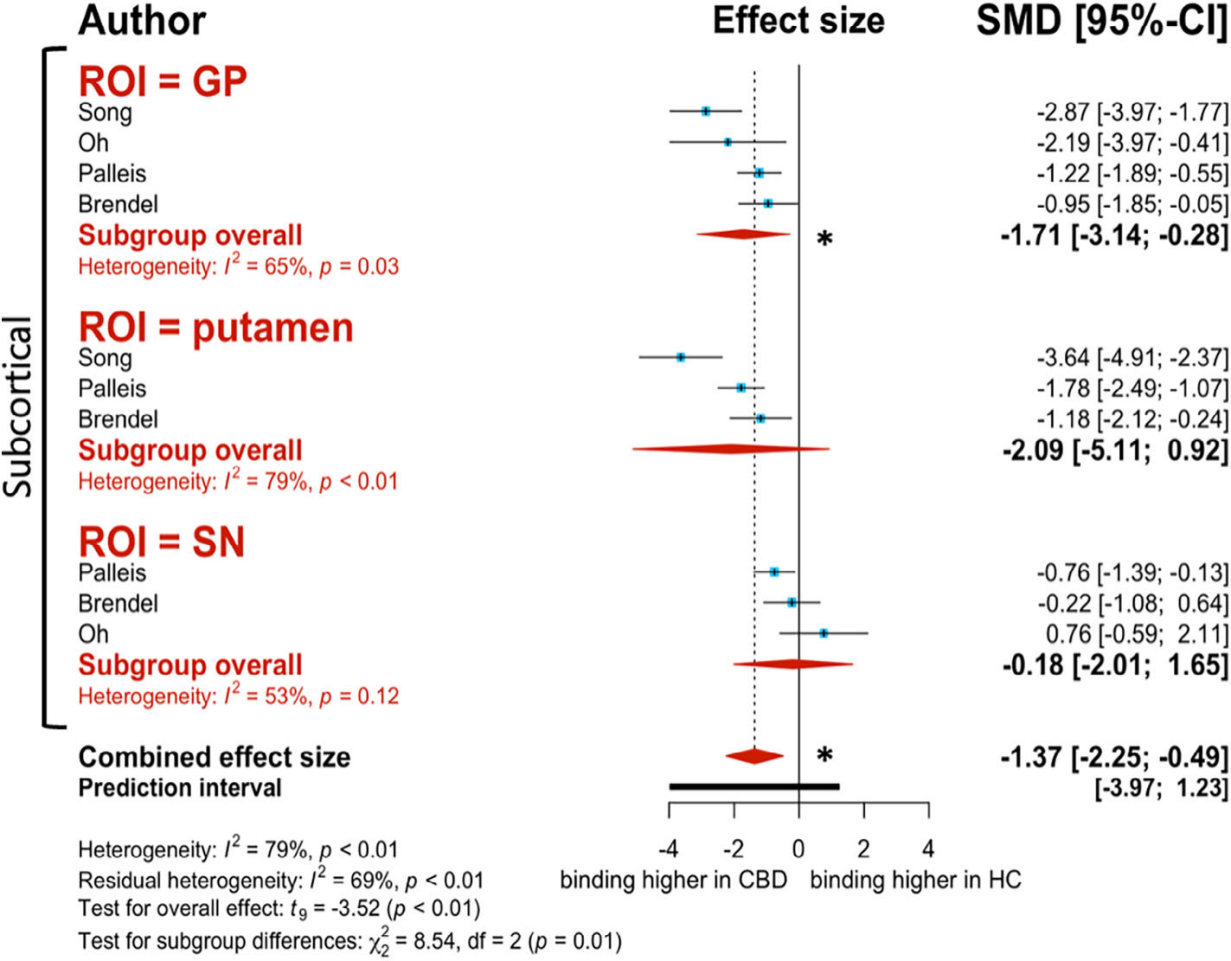
Forest plot of differences in second‐generation tracer [^18^F]PI‐2620 binding to corticobasal degeneration (CBD) patients versus healthy controls (HC). Red diamonds represent subgroup analysis based on region of interest (ROI). Blue data points represent individual study effect sizes. The * represents statistical significance (*P* < 0.05).

Overall, there was significantly higher [^18^F]PI‐2620 binding in *CBD patients over HC* (*g* = −1.37, 95% CI: −2.25 to −0.49, *P* = 0.007; Fig. 4). All ROI subgroups displayed this same trend, although the GP is the only ROI that presents with significantly higher binding in CBD patients over HC (*g* = −1.71, 95% CI: −3.14 to −0.28, *I* ^2^ = 65.4%; Fig. 4). Although there were no significant effect moderators for this analysis, there was a strong correlation for publication year. More recent publications demonstrated higher binding to CBD patients over HC (estimate = −1.234, 95% CI: −2.824 to 0.355, *P* = 0.111).

### Parkinson's Disease

#### First‐Generation Tracer

Overall, [^18^F]AV‐1451 binding to the ROIs in PD patients was unremarkable. When compared to HC, tracer binding was higher in HC than PD patients (*g* = 0.25, 95% CI: −0.0006 to 0.50, *P* = 0.05), although the results were not significant overall. Through subgroup analysis, we found that there were no significant differences in tracer binding for any of the ROIs, except for the SN. [^18^F]AV‐1451 binding was significantly higher in HC than PD patients in the SN (*g* = 0.94, 95% CI: 0.56 to 1.33, *I* ^2^ = 0.0%). Percentage of PD females was an effect moderator for [^18^F]AV‐1451 binding. A higher percentage of PD females in the studies assessed correlated with lower binding values for PD patients relative to HC (estimate = 0.021, 95% CI: 0.006 to 0.037, *P* = 0.008).

#### Second‐Generation Tracer

An opposite trend was seen with the second‐generation tracer [^18^F]PM‐PBB3 when compared to [^18^F]AV‐1451. With this tracer we observed higher binding in PD patients over HC (*g* = −0.16, 95% CI: −0.41 to 0.08, *P* = 0.16). Although the results here were not significant, the ROIs that displayed this trend were the putamen (*g* = −0.41, 95% CI: −0.84 to 0.02, *I* ^2^ = 0.0%) and temporal lobe (*g* = −0.14, 95% CI: −0.80 to 0.52, *I* ^2^ = 0.0%). Age of PD participants was an effect moderator for [^18^F]PM‐PBB3 binding. Among all studies assessed, advanced PD age was associated with increased binding to PD patients over HC (estimate −0.051, 95% CI: −0.092 to −0.011, *P* = 0.016).

## Discussion

Our first main finding of this meta‐analysis was that all tau PET tracers assessed demonstrate trends towards higher binding in AP patients (i.e., PSP, and CBD) when compared to HC and PD. This is a trend that we would expect to see because pathological tau accumulates in patients with these diseases.^40^, ^41^, ^42^ Consistent with the literature, we found that the second‐generation tracer [^18^F]PI‐2620 outperforms first‐generation tracers for both PSP and CBD.^33^, ^34^, ^35^, ^37^, ^43^ The larger effect sizes and reduced heterogeneity (i.e. lower I^2^ values) in the analyses when using this tracer demonstrates increased reliability for this data and supports the use of [^18^F]PI‐2620 as a potential imaging biomarker for these tauopathies. Interestingly, when comparing PD patients to HC, we found opposing trends for the first‐generation tracer [^18^F]AV‐1451 and the second‐generation tracer [^18^F]PM‐PBB3. The trend towards higher binding to HC with [^18^F]AV‐1451 seems to be predominantly driven by binding to the SN. This tracer is reported to observe extensive off‐target binding to the SN, neuromelanin, and MAO‐B.^13^ This off‐target binding could be the main reason for the trend observed with this tracer but there is also the issue of extensive SN degeneration in late‐stage PD patients.^44^, ^45^ Data for [^18^F]PM‐PBB3 tracer binding to the SN was insufficient for the present model, but recent literature states that this tracer has mediated many of the off‐target binding issues observed with first‐generation tracers, including off‐target binding to the SN.^46^, ^47^

Our second finding was that when comparing parkinsonisms head‐to‐head, there was significantly higher binding to PSP patients over PD when using the first‐generation tracer [^18^F]AV‐1451. There was only sufficient data to conduct this group comparison with one tracer but these results show that despite the limitations of using [^18^F]AV‐1451, this tracer is still able to differentiate PSP from PD patients using tau binding values. In particular, the GP is the ROI that presents with the largest SMD in tracer binding and this has been one of the regions cited in the literature as the most severely affected in PSP patients.^48^ Although there was insufficient data to conduct this group comparisons with the other tracers, recent studies have provided evidence that some second‐generation tracers can effectively distinguish PD from APs. In their large‐scale, multi‐site study, Brendel et al^37^ provided evidence for the use of [^18^F]PI‐2620 in differential diagnosis of PSP from other neurodegenerative diseases, including PD. Here, the researchers cited the GP internus (GPi) as the region with the strongest differences between PSP and other neurodegenerative diseases.

Our third finding was that the ROIs that consistently presented with the largest effect sizes in the pathological groups were the subcortical structures. Reviews of AP neuropathology have cited the basal ganglia as the most severely affected region for both PSP and CBD. This part of the brain observes extensive neurodegeneration and tau accumulation throughout disease progression. In our analysis, we observe large effect size values particularly for the GP. The main function of the GP is the control of voluntary movement; that is, it facilitates the control of conscious and voluntary movements.^49^, ^50^, ^51^ Hence, the extensive accumulation of tau in this region, particularly for PSP and CBD patients explains the complex motor symptoms observed in these patients. The symptomology for APs is quite heterogenous and this is thought to be a direct link to differential patterns of tau accumulation. Although the patterns of tau deposits may vary, the GP seems to be one of the most consistently affected regions and could be a good ROI to serve as a means for differential diagnosis of PSP and CBD from other parkinsonisms.

Finally, our meta‐regression analyses revealed interesting points of consideration for future in vivo studies with these tau PET tracers. The effect moderator patterns for second‐generation tracers were in line with what we expected to see. For example, with longer PD disease durations, we expected to see a stronger correlation with increased binding to PD over HC and this was indeed the case with [^18^F]PM‐PBB3. Similarly, with more experience applying second‐generation tracers to this patient population it is expected that a trend in the data arises. Thus, the association between more recent publications and higher binding to CBD patients over HC with the [^18^F]PI‐2620 tracer is in line with our expectations. With regard to the first‐generation tracers' meta‐regression analyses, the [^18^F]AV‐1451 PSP‐PD group comparison revealed a significant association between PSP duration and binding to PSP over PD. Tau accumulates with advanced age under normal physiological conditions and especially with late‐stage parkinsonism patients, but this build‐up is evidently heightened in patients with late‐stage PSP.

Interestingly, we saw an unexpected trend for the [^18^F]THK‐5351 PSP‐HC and [^18^F]AV‐1451 CBD‐HC group comparisons. Here, we observed misleading positive correlation coefficients associated with disease duration, suggesting that with increased disease duration there was a trend towards decreased binding to the pathological groups. To our knowledge, there are no longitudinal studies with [^18^F]THK‐5351 PET in APs, but there is at least one conducted with [^18^F]AV‐1451 PET.^17^ In this study, researchers reported [^18^F]AV‐1451 measures to increase over time, in line with disease duration, although these changes did not correlate with changes on the PSPRS. Therefore, we suggest that these unexpected correlations are potential artifacts and may be explained by the inconsistencies and off‐target binding issues observed with first‐generation tracers when applied to patients with APs.

As stated, tau accumulation tends to build up with advanced age which is referred to as primary age‐related tauopathy (or PART). For this reason, we included age as a moderator in our meta‐regression analysis. Typically, we would expect studies with older participants to have higher tau PET binding values and this is what we found with the [^18^F]AV‐1451 CBD‐HC and [^18^F]PM‐PBB3 PD‐HC group comparisons. For [^18^F]AV‐1451, advanced CBD age was associated with higher binding to CBD participants over HC. Similarly, the [^18^F]PM‐PBB3 tracer demonstrated that advanced PD age was associated with higher binding to PD patients over HC. Although [^18^F]AV‐1451's binding patterns in relation to patient age are consistent for the CBD‐HC comparison, we observed an opposite pattern arise for the PSP‐PD comparison. Here, contrary to what we expected, advanced PSP age was associated with decreased binding to PSP relative to PD. Typically, we would expect the opposite pattern due to the neuropathologic nature of PSP and the increased accumulation of tau in older PSP patients. As already noted above, these unexpected correlations seemed to be present solely for the first‐generation tracers which may, in part, be explained by their instability and reduced capacity for application with 4R‐tauopathies like PSP and CBD.

Overall, the percentage of female participants was a significant effect moderator for the [^18^F]AV‐1451 PD‐HC group comparison. The percentage of PD females in the [^18^F]AV‐1451 PD‐HC comparison correlated with decreased binding to PD relative to HC. The correlation coefficient for this effect was quite low (estimate = 0.021, 95% CI: 0.006 to 0.037, *P* = 0.008), indicating a weak correlation. Additionally, it has been reported that there are sex differences in off‐target binding with [^18^F]AV‐1451, where women demonstrate higher off‐target binding particularly to the skull/meninges.^52^ No such study has been conducted using the other tracers but since there are some inconsistencies here, it would be interesting to explore this phenomenon further in future work.

### Limitations and Future Directions

Given the variations in methodological factors between the included studies, there was moderate between‐study inconsistencies observed throughout the analyses conducted for the present study. Some of these variations arose due to differences in PET scanners, PET cameras and resolutions, voxel positioning, and tracer uptake calculation methods. Additional, non‐methodological factors to consider may include the clinical characteristics of the participants due to the heterogeneous nature of the diseases involved.

For the purpose of this meta‐analysis, we combined all studies and selected ROI as the basis for our subgroup analysis. Given the brain regional variability in tau burden cited in the literature this was assessed as the most important grouping factor. However, there is the possibility that the methodological variability discussed here could have implications for the results of this study and this is something that should be accounted for when meta‐analyzing future imaging studies.

The present study demonstrates the great strides and improvements that have taken place with the progression from first‐ to second‐generation tau PET tracers. The results of our meta‐analysis demonstrate this through the larger effect sizes and reduced heterogeneity (ie, lower I^2^ values) observed with the use of second‐generation tracers. One limitation of this study is the limited sample size of studies that met our inclusion criteria for the meta‐analytic model. Future tau PET imaging studies would benefit from following a standardized reporting schema, allowing for imaging data availability and future quantitative comparisons.

In recent years, there has been growing use and progression to the use of new second‐generation tau PET tracers in patients with APs. Many of these studies are still in progress and it would be interesting for future work to look at head‐to‐head comparisons of the second‐generation tracers discussed here against some of those currently being applied in APs. These include tracers like [^18^F]MK‐6240 which is now being tested in patients with APs.^53^

## Conclusion

Overall, we found that the second‐generation tracer [^18^F]PI‐2620 was the most optimal of those included in this study for imaging tau in PSP and CBD. The second‐generation tracer [^18^F]PM‐PBB3 was also able to effectively differentiate PD patients from HC using tau binding patterns. In terms of differential diagnostic capacity of tau PET tracers, the first‐generation tau tracer [^18^F]AV‐1451 presents with favorable binding to PSP over PD patients, although these results have a large amount of heterogeneity, indicated by high I^2^ values. Given the significant between‐study heterogeneity for first‐generation tracers, the results of the present study support the shift towards the application of second‐generation tau PET tracers in the exploration of differential diagnostic biomarkers for PSP and CBD.

## Author Roles

(1) Research project: A. Conception, B. Organization, C. Execution; (2) Statistical analysis: A. Design, B. Execution, C. Review and critique; (3) Manuscript: A. Writing of the first draft, B. Review and critique.

A.M.M.: 1A, 1B, 1C, 2A, 2B, 3A, 3B

R.C.: 2A, 2C, 3B

A.G.‐G.: 2A, 2C, 3B

S.L.M.: 2C, 3B

C.U.: 2C, 3B

A.P.S.: 1A, 1B, 2A, 2C, 3B

## Disclosures

**Ethical Compliance Statement:** The authors confirm that the approval of an institutional review board was not required for this work. The authors confirm that patient consent was not required for this work. We confirm that we have read the Journal's position on issues involved in ethical publication and affirm that this work is consistent with those guidelines.

**Funding Sources and Conflicts of Interest:** This work was supported by Canadian Institutes of Health Research (CIHR) (PJT‐ 173540). A.P.S. is supported by the Krembil‐Rossy Chair program. Antonio Strafella was a consultant for Hoffman La Roche; in the past, he received honoraria from GE Health Care Canada LTD, Hoffman La Roche.

**Financial Disclosures for the Previous 12 Months:** There were no financial disclosures to list for the previous 12 months.

## Supporting information


**Table S1.** Modified Newcastle‐Ottawa Scale (NOS) ratings for included studies

**Table S2.** Summary of studies included in meta‐analysis
