# Supplementary material for: Tau in Atypical Parkinsonisms: A Meta‐Analysis of in Vivo PET Imaging Findings
Source: Mov Disord Clin Pract. 2023 Sep 29;10(12):1725–37. doi: 10.1002/mdc3.13885 (PMC10715372; doi:10.1002/mdc3.13885)
Supplement: Supplementary file 1 — Table S1. Modified Newcastle‐Ottawa Scale (NOS) ratings for included studies [file MDC3-10-1725-s001.docx]

Table S1. Modified Newcastle-Ottawa Scale (NOS) ratings for included studies

| Study | Representativeness of patient cohorts | Selection and comparability of the control cohort | Diagnostic criteria used for PD/PSP/CBD | PET/scanning and radiochemistry | PET image analysis | Disease characteristics described | Total |
| --- | --- | --- | --- | --- | --- | --- | --- |
| AV-1451 | | | | | | | |
| Hansen 2016 | 0 | X | X | X | X | X | 5 |
| Cho 2017 | 0 | X | X | X | X | X | 5 |
| Gomperts 2016 | 0 | X | X | X | X | X | 5 |
| Holland 2021 | 0 | X | X | X | X | X | 5 |
| Whitwell 2019 | 0 | X | X | X | X | X | 5 |
| Smith 2016 | 0 | X | X | X | X | X | 5 |
| Ossenkoppele 2018 | X | X | X | X | X | X | 6 |
| Coakeley 2017 | 0 | X | X | X | X | X | 5 |
| Schonhaut 2017 | 0 | X | X | X | X | X | 5 |
| Li 2021 | X | X | X | X | X | X | 6 |
| Niccolini 2018 | 0 | X | X | X | X | X | 5 |
| Smith 2017 | 0 | 0 | X | X | X | X | 4 |
| Tsai 2019 | 0 | X | X | X | X | 0 | 4 |
| Winer 2018 | 0 | X | X | X | X | X | 5 |
| Hansen 2017 | 0 | X | X | X | X | X | 5 |
| PI-2620 | | | | | | | |
| Song 2021 | 0 | X | X | X | X | 0 | 4 |
| Messerschmidt 2022 | 0 | X | X | X | X | X | 5 |
| Oh 2020 | X | X | X | X | X | 0 | 5 |
| Palleis 2021 | 0 | X | X | X | X | X | 5 |
| Song 2021 | 0 | X | X | X | X | 0 | 4 |
| Brendel 2020 | X | X | X | X | X | X | 6 |
| PM-PBB3 | | | | | | | |
| Li 2021 | 0 | X | X | X | X | X | 5 |
| Liu 2022 | 0 | X | X | X | X | X | 5 |
| Tang 2022 | 0 | X | X | X | X | X | 5 |
| THK-5351 | | | | | | | |
| Brendel 2018 | 0 | X | X | X | X | X | 5 |
| Ezura 2021 | 0 | X | X | X | X | X | 5 |
| Hsu 2020 | 0 | X | X | X | X | X | 5 |
| Ishiki 2017 | 0 | X | X | X | X | 0 | 4 |
| Ng 2019 | 0 | 0 | X | X | X | X | 4 |
