# Supplementary material for: Tau in Atypical Parkinsonisms: A Meta‐Analysis of in Vivo PET Imaging Findings
Source: Mov Disord Clin Pract. 2023 Sep 29;10(12):1725–37. doi: 10.1002/mdc3.13885 (PMC10715372; doi:10.1002/mdc3.13885)
Supplement: Supplementary file 2 — Table S2. Summary of studies included in meta‐analysis [file MDC3-10-1725-s002.docx]

Table S2. Summary of studies included in meta-analysis

| **Study** | **Method** | **Tracer** | **Injected dose**  **(Mbq)** | **Scan duration**  **(min)** | **Scanner^A^** | **Analysis method^B^** |
| --- | --- | --- | --- | --- | --- | --- |
| Hansen 2016 | PET | [^18^F]AV-1451 | 300-370 | 40 | ECAT HRRT; CTI/Siemens | SUVR |
| Cho 2017 | PET | [^18^F]AV-1451 | 283 | 20 | Siemens Biograph mCT PET | SUVR |
| Gomperts 2016 | PET | [^18^F]AV-1451 | 370 | 20 | CTI ECAT HR+; Siemens | SUVR |
| Holland 2021 | PET | [^18^F]AV-1451 | - | 90 | GE SIGNA PET/MR | BP |
| Whitwell 2019 | PET | [^18^F]AV-1451 | 370 | 20 | GE PET/CT scanner | SUVR |
| Smith 2016 | PET | [^18^F]AV-1451 | 372 | 40 | GE Discovery 690 PET/CT | SUVR |
| Ossenkoppele 2018 | PET | [^18^F]AV-1451 | 370 (BioFINDER and UCSF) or 280 (Seoul) | 60 | Siemens Biograph mCT PET/CT (Seoul), GE Discovery 690 PET (BioFINDER), Siemens Biograph 6 Truepoint PET/CT (Berkeley), GE Discovery VCT PET/CT (UCSF) | SUVR |
| Coakeley 2017 | PET | [^18^F]AV-1451 | - | 90 | PET/CT Siemens-Biograph HiRez XVI | SUVR |
| Schonhaut 2017 | PET | [^18^F]AV-1451 | 370 | 20 | - | SUVR |
| Li 2021 | PET | [^18^F]AV-1451 | 370 | 20 | Siemens Biograph mCT PET/CT | SUVR |
| Niccolini 2018 | PET | [^18^F]AV-1451 | - | 20 | - | SUVR |
| Smith 2017 | PET | [^18^F]AV-1451 | - | 40 | GE Discovery 690 PET/CT | SUVR |
| Tsai 2019 | PET | [^18^F]AV-1451 | 370 | 20 | Siemens Biograph Truepoint PET/CT | SUVR |
| Winer 2018 | PET | [^18^F]AV-1451 | - | 20 | Siemens Biograph 6 PET/CT | SUVR |
| Hansen 2017 | PET | [^18^F]AV-1451 | 300 | 40 | ECAT HRRT; CTI/Siemens | SUVR |
| Brendel 2018 | PET | [^18^F]THK-5351 | 183 | 40 | GE Discovery 690 PET/CT scanner | SUVR |
| Ezura 2021 | PET | [^18^F]THK-5351 | 185 | 20 | Shimadzu Eminence STARGATE scanner | SUVR |
| Hsu 2020 | PET | [^18^F]THK-5351 | 378 | 60 | Siemens Biograph mCT 16 scanner | SUVR |
| Ishiki 2017 | PET | [^18^F]THK-5351 | 185 | 60 | Shimadzu Eminence STARGATE scanner | SUVR |
| Ng 2019 | PET | [^18^F]THK-5351 | 255.3 | 70 | Siemens High Resolution Research Tomograph (HRRT) | SUV |
| Song 2021 | PET | [^18^F]PI-2620 | 217 | 60 | Siemens Biograph True point 64 PET/CT (Munich), Siemens ECAT EXACT HR+ (New Haven), Philips Gemini TF 64 PET/CT (Melbourne) | SUVR |
| Messerschmidt 2022 | PET | [^18^F]PI-2620 | 217 | 60 | Siemens Biograph True point 64 PET/CT (Munich), Siemens ECAT EXACT HR+ (New Haven), Philips Gemini TF 64 PET/CT (Melbourne) | SUVR |
| Oh 2020 | PET | [^18^F]PI-2620 | 259 | 30 | GE Discovery 690, 710, and 690 Elite PET/CT | SUVR |
| Palleis 2021 | PET | [^18^F]PI-2620 | 176-334 | 60 | - | DVR |
| Song 2021 | PET | [^18^F]PI-2620 | 168-334 | 40 | Siemens Biograph True point 64 PET/CT (Munich), Biograph mMR hybrid PET/MR (Leipzig), Siemens mCT (Cologne), Siemens ECAT EXACT HR+ (New Haven), Philips Gemini TF 64 PET/CT (Melbourne) | DVR |
| Brendel 2020 | PET | [^18^F]PI-2620 | 168-334 | 60 | Siemens Biograph True point 64 PET/CT (Munich), Biograph mMR hybrid PET/MR (Leipzig), Siemens mCT (Cologne), Siemens ECAT EXACT HR+ (New Haven), Philips Gemini TF 64 PET/CT (Melbourne) | DVR |
| Li 2021 | PET | [^18^F]PM-PBB3 | 370 | 20 | Siemens mCT Flow PET/CT scanner (Siemens, Erlangen, Germany) | SUVR |
| Liu 2022 | PET | [^18^F]PM-PBB3 | 370 | 20 | mCT Flow PET/computed tomography; Siemens Healthcare GmbH, Erlangen, Germany | SUVR |
| Tang 2022 | PET | [^18^F]PM-PBB3 | 185-259 | 20 | Siemens mCT Flow PET/CT scanner (Siemens, Erlangen, Germany) | SUVR |

^A^ Scanner models written as they were reported in the original articles

^B^ SUVR = standardized uptake value ratio, BP = binding potential, SUV = standardized uptake value
